# Supplementary material for: The Impact of Free and Added Sugars on Cognitive Function: A Systematic Review and Meta-Analysis
Source: Nutrients. 2023 Dec 25;16(1):75. doi: 10.3390/nu16010075 (PMC10780393; doi:10.3390/nu16010075)
Supplement: Supplementary file 1 [file nutrients-16-00075-s001.zip › Free_Sugars_Supplementary File S1_v2.pdf]

Supplementary Table S1: Results of searches conducted on 30th August 2023.

PubMed		
**Search #**	**Search terms**	**Items found**
#1	"cognition"[MeSH Terms] OR "executive function"[MeSH Terms]	201,156
#2	"cognit*"[Title/Abstract] OR "executive function*"[Title/Abstract] OR "brain function*"[Title/Abstract] OR "memory"[Title/Abstract] OR "decision making"[Title/Abstract] OR "inhibitory control"[Title/Abstract] OR "mental planning"[Title/Abstract] OR "psychomotor speed"[Title/Abstract] OR "psycho-motor speed"[Title/Abstract]	874,680
#3	#1 OR #2	955,249
#4	"sugars"[MeSH Terms]	436,872
#5	"sugar*"[Title/Abstract] OR "carbohydrate*"[Title/Abstract] OR "sucrose"[Title/Abstract] OR "fructose"[Title/Abstract] OR "glucose"[Title/Abstract]	820,736
#6	#4 OR #5	966,989
#7	#3 AND #6	15,817
#8	"stroke"[MeSH Terms] OR "neoplasms"[MeSH Terms] OR "schizophrenia"[MeSH Terms]	3,982,840
#9	"diabetic"[Title/Abstract] OR "cancer*"[Title/Abstract] OR "neoplasm*"[Title/Abstract] OR "adenoma"[Title/Abstract] OR "chemotherapy"[Title/Abstract] OR "schizophreni*"[Title/Abstract] OR "pesticide"[Title/Abstract] OR "bumblebee*"[Title/Abstract] OR "honeybee*"[Title/Abstract] OR "honey bee*"[Title/Abstract] OR "encephalopathy"[Title/Abstract] OR "earthworm"[Title/Abstract] OR "drosophila"[Title/Abstract] OR "covid"[Title/Abstract] OR "covid-19"[Title/Abstract] OR "HIV"[Title/Abstract] OR "opioid*"[Title/Abstract] OR "alcohol"[Title/Abstract] OR "cocaine"[Title/Abstract] OR "methylphenidate"[Title/Abstract] OR "NSAID"[Title/Abstract] OR "hypoxi*"[Title/Abstract] OR "atherosclerosis"[Title/Abstract] OR "apnea"[Title/Abstract] OR "ischemi*"[Title/Abstract] OR "ischaemi*"[Title/Abstract] OR "alopecia"[Title/Abstract] OR "fibromyalgia"[Title/Abstract] OR "liver disease"[Title/Abstract] OR "epilep*"[Title/Abstract] OR "herpes"[Title/Abstract] OR "anorexi*"[Title/Abstract] OR "bulimi*"[Title/Abstract] OR "parkinson*"[Title/Abstract] OR "huntington*"[Title/Abstract] OR "stroke"[Title/Abstract] OR "tourette*"[Title/Abstract] OR "myocardial infarction"[Title/Abstract] OR "soil"[Title/Abstract] OR "multiple sclerosis"[Title/Abstract] OR "bipolar"[Title/Abstract] OR "autism"[Title/Abstract] OR "autistic"[Title/Abstract] OR "ADHD"[Title/Abstract] OR "brain injury"[Title/Abstract] OR "mild cognitive impairment"[Title/Abstract]	5,903,114
#10	#8 OR #9	7,729,023
#11	#7 NOT #10	9,825
**Embase**		
**Search #**	**Search terms**	**Items found**
#1	cognition'/exp	2,817,625
#2	executive function'/exp	50,107

#3	cognit*:ab,ti OR 'executive function*':ab,ti OR 'brain function*':ab,ti OR memory:ab,ti OR 'mental planning':ab,ti OR 'decision making':ab,ti OR 'inhibitory control':ab,ti OR 'psychomotor speed':ab,ti OR 'psycho-motor speed':ab,ti	1,139,922
#4	#1 OR #2 OR #3	3,258,609
#5	sugar'/exp	31,015
#6	sugar*:ab,ti OR carbohydrate*:ab,ti OR glucose:ab,ti OR fructose:ab,ti OR sucrose:ab,ti	1,033,373
#7	#5 OR #6	1,037,693
#8	#4 AND #7	46,922
#9	'schizophrenia'/exp	208,527
#10	diabetes mellitus'/exp	1,163,949
#11	cerebrovascular accident'/exp	390,372
#12	diabetic:ab,ti OR cancer*:ab,ti OR neoplasm*:ab,ti OR adenoma:ab,ti OR chemotherapy:ab,ti OR pesticide:ab,ti OR bumblebee*:ab,ti OR honeybee*:ab,ti OR 'honey bee*':ab,ti	3,855,750
#13	encephalopathy:ab,ti OR earthworm:ab,ti OR drosophila:ab,ti OR covid:ab,ti OR 'covid 19':ab,ti OR hiv:ab,ti OR opioid:ab,ti OR alcohol*:ab,ti OR cocaine:ab,ti OR methylphenidate:ab,ti OR nsaid:ab,ti	1,554,609
#14	hypoxi*:ab,ti OR atherosclerosis:ab,ti OR apnea:ab,ti OR ischemi*:ab,ti OR ischaemi*:ab,ti OR alopecia:ab,ti OR fibromyalgia:ab,ti OR 'liver disease':ab,ti OR epilep*:ab,ti OR herpes:ab,ti OR anorexi*:ab,ti OR bulimi*:ab,ti	1,572,039
#15	parkinson*:ab,ti OR huntington*:ab,ti OR stroke:ab,ti OR tourette*:ab,ti OR 'myocardial infarction':ab,ti OR soil:ab,ti OR 'multiple sclerosis':ab,ti OR bipolar:ab,ti OR autism:ab,ti OR autistic:ab,ti OR adhd:ab,ti	1,440,534
#16	schizophreni*:ab,ti OR 'mild cognitive impairment':ab,ti OR 'brain injury':ab,ti	317,469
#17	#9 OR #10 OR #11 OR #12 OR #13 OR #14 OR #15 OR #16	8,713,247
#18	#8 NOT #17	
	**Cinahl**	
S1	(MH "Cognition+")	75,951
S2	(MH "Executive Function")	6,049
S3	TI cognit* OR TI "executive function*" OR TI "brain function*" OR TI memory OR TI "decision making" OR TI "inhibitory control" OR TI "mental planning" OR TI "psychomotor speed" OR TI "psycho-motor speed"	102,970
S4	AB cognit* OR AB "executive function*" OR AB "brain function*" OR AB memory OR AB "decision making" OR AB "inhibitory control" OR AB "mental planning" OR AB "psychomotor speed" OR AB "psycho-motor speed"	243,816
S5	S1 OR S2 OR S3 OR S4	309,676
S6	TI sugar* OR TI fructose OR TI sucrose OR TI glucose OR TI carbohydrate*	33,045
S7	AB sugar* OR AB fructose OR AB sucrose OR AB glucose OR AB carbohydrate*	93,842
S8	S6 OR S7	106,811
S9	S5 AND S8	3,272
S10	(MH "Stroke+")	77,673

S11	(MH "Neoplasms+")	631,127
S12	(MH "Schizophrenia+")	27,889
S13	TI diabetic OR TI cancer* OR TI neoplasm* OR TI adenoma OR TI chemotherapy OR TI pesticide OR TI bumblebee* OR TI honeybee* OR TI "honey bee*"	381,224
S14	AB diabetic OR AB cancer* OR AB neoplasm* OR AB adenoma OR AB chemotherapy OR AB pesticide OR AB bumblebee* OR AB honeybee* OR AB "honey bee*"	415,434
S15	TI encephalopathy OR TI earthworm OR TI drosophila OR TI covid OR TI covid-19 OR TI hiv OR TI opioid OR TI alcohol* OR TI cocaine OR TI methylphenidate OR TI NSAID	219,623
S16	AB encephalopathy OR AB earthworm OR AB drosophila OR AB covid OR AB covid-19 OR AB hiv OR AB opioid OR AB alcohol* OR AB cocaine OR AB methylphenidate OR AB NSAID	255,617
S17	TI hypoxi* OR TI atherosclerosis OR TI apnea OR TI ischemi* OR TI ischaemi* OR TI alopecia OR TI fibromyalgia OR TI "liver disease" OR TI epilep* OR TI herpes OR TI anorexi* OR TI bulimi*	93,394
S18	AB hypoxi* OR AB atherosclerosis OR AB apnea OR AB ischemi* OR AB ischaemi* OR AB alopecia OR AB fibromyalgia OR AB "liver disease" OR AB epilep* OR AB herpes OR AB anorexi* OR AB bulimi*	152,875
S19	TI parkinson* OR TI huntington* OR TI stroke OR TI tourette* OR TI "myocardial infarction" OR TI soil OR TI "multiple sclerosis" OR TI bipolar OR TI autism OR TI autistic OR TI ADHD	167,823
S20	AB parkinson* OR AB huntington* OR AB stroke OR AB tourette* OR AB "myocardial infarction" OR AB soil OR AB "multiple sclerosis" OR AB bipolar OR AB autism OR AB autistic OR AB ADHD	199,303
S21	TI schizophreni* OR TI "mild cognitive impairment" OR TI "brain injury"	41,453
S22	AB schizophreni* OR AB "mild cognitive impairment" OR AB "brain injury"	52,683
S23	S10 OR S11 OR S12 OR S13 OR S14 OR S15 OR S16 OR S17 OR S18 OR S19 OR S20 OR S21 OR S22	1,607,686
S24	S9 NOT S23	2,209
**PsychArticles**		
S1	DE cognition	6,157
S2	DE executive function	1,142
S3	TI cognit* OR TI "executive function*" OR TI "brain function*" OR TI memory OR TI "decision making" OR TI "inhibitory control" OR TI "mental planning" OR TI "pscyhomotor speed" OR TI "psycho-motor speed"	14,834
S4	AB cognit* OR AB "executive function*" OR AB "brain function*" OR AB memory OR AB "decision making" OR AB "inhibitory control" OR AB "mental planning" OR AB "pscyhomotor speed" OR AB "psycho-motor speed"	35,963
S5	S1 OR S2 OR S3 OR S4	39,571
S6	DE sugars	127
S7	TI sugar* OR TI fructose OR TI sucrose OR TI glucose OR TI carbohydrate*	192
S8	AB sugar* OR AB fructose OR AB sucrose OR AB glucose OR AB carbohydrate*	846

S9	S6 OR S7 OR S8	854
S10	S5 AND S9	113
S11	DE schizophrenia	4,594
S12	DE stroke	73
S13	DE neoplasms	742
S14	TI diabetic OR TI cancer* OR TI neoplasm* OR TI adenoma OR TI chemotherapy OR TI pesticide OR TI bumblebee* OR TI honeybee* OR TI "honey bee*"	830
S15	AB diabetic OR AB cancer* OR AB neoplasm* OR AB adenoma OR AB chemotherapy OR AB pesticide OR AB bumblebee* OR AB honeybee* OR AB "honey bee*"	1,450
S16	TI encephalopathy OR TI earthworm OR TI drosophila OR TI covid OR TI covid-19 OR TI hiv OR TI opioid OR TI alcohol* OR TI cocaine OR TI methylphenidate OR TI NSAID	4,755
S17	AB encephalopathy OR AB earthworm OR AB drosophila OR AB covid OR AB covid-19 OR AB hiv OR AB opioid OR AB alcohol* OR AB cocaine OR AB methylphenidate OR AB NSAID	7,975
S18	TI hypoxi* OR TI atherosclerosis OR TI apnea OR TI ischemi* OR TI ischaemi* OR TI alopecia OR TI fibromyalgia OR TI "liver disease" OR TI epilep* OR TI herpes OR TI anorexi* OR TI bulimi*	508
S19	AB hypoxi* OR AB atherosclerosis OR AB apnea OR AB ischemi* OR AB ischaemi* OR AB alopecia OR AB fibromyalgia OR AB "liver disease" OR AB epilep* OR AB herpes OR AB anorexi* OR AB bulimi*	1,072
S20	TI parkinson* OR TI huntington* OR TI stroke OR TI tourette* OR TI "myocardial infarction" OR TI soil OR TI "multiple sclerosis" OR TI bipolar OR TI autism OR TI autistic OR TI ADHD	1,664
S21	AB parkinson* OR AB huntington* OR AB stroke OR AB tourette* OR AB "myocardial infarction" OR AB soil OR AB "multiple sclerosis" OR AB bipolar OR AB autism OR AB autistic OR AB ADHD	3,274
S22	TI schizophreni* OR TI "mild cognitive impairment" OR TI "brain injury"	3,713
S23	AB schizophreni* OR AB "mild cognitive impairment" OR AB "brain injury"	5,672
S24	S11 OR S12 OR S13 OR S14 OR S15 OR S16 OR S17 OR S18 OR S19 OR S20 OR S21 OR S22 OR S23	19,514
S25	S10 NOT S24	100
	**PsychINFO**	
S1	DE "Cognition"	82,268
S2	DE "Executive Function"	18,684
S3	TI cognit* OR TI "executive function*" OR TI "brain function*" OR TI memory OR TI "decision making" OR TI "inhibitory control" OR TI "mental planning" OR TI "psychomotor speed" OR TI "psycho-motor speed"	265,392
S4	AB cognit* OR AB "executive function*" OR AB "brain function*" OR AB memory OR AB "decision making" OR AB "inhibitory control" OR AB "mental planning" OR AB "psychomotor speed" OR AB "psycho-motor speed"	725,035
S5	S1 OR S2 OR S3 OR S4	775,081
S6	DE "Sugars"	2,469

S7	TI sugar* OR TI fructose OR TI sucrose OR TI glucose OR TI carbohydrate*	6,543
S8	AB sugar* OR AB fructose OR AB sucrose OR AB glucose OR AB carbohydrate*	31,762
S9	S6 OR S7 OR S8	32,442
S10	S5 AND S9	4,638
S11	DE "Schizophrenia"	103,249
S12	DE "Cerebrovascular Accidents"	23,290
S13	DE "Neoplasms"	44,009
S14	TI diabetic OR TI cancer* OR TI neoplasm* OR TI adenoma OR TI chemotherapy OR TI pesticide OR TI bumblebee* OR TI honeybee* OR TI "honey bee*"	45,726
S15	AB diabetic OR AB cancer* OR AB neoplasm* OR AB adenoma OR AB chemotherapy OR AB pesticide OR AB bumblebee* OR AB honeybee* OR AB "honey bee*"	79,374
S16	TI encephalopathy OR TI earthworm OR TI drosophila OR TI covid OR TI covid-19 OR TI hiv OR TI opioid OR TI alcohol* OR TI cocaine OR TI methylphenidate OR TI NSAID	146,227
S17	AB encephalopathy OR AB earthworm OR AB drosophila OR AB covid OR AB covid-19 OR AB hiv OR AB opioid OR AB alcohol* OR AB cocaine OR AB methylphenidate OR AB NSAID	255,388
S18	TI hypoxi* OR TI atherosclerosis OR TI apnea OR TI ischemi* OR TI ischaemi* OR TI alopecia OR TI fibromyalgia OR TI "liver disease" OR TI epilep* OR TI herpes OR TI anorexi* OR TI bulimi*	52,048
S19	AB hypoxi* OR AB atherosclerosis OR AB apnea OR AB ischemi* OR AB ischaemi* OR AB alopecia OR AB fibromyalgia OR AB "liver disease" OR AB epilep* OR AB herpes OR AB anorexi* OR AB bulimi*	99,718
S20	TI parkinson* OR TI huntington* OR TI stroke OR TI tourette* OR TI "myocardial infarction" OR TI soil OR TI "multiple sclerosis" OR TI bipolar OR TI autism OR TI autistic OR TI ADHD	129,206
S21	AB parkinson* OR AB huntington* OR AB stroke OR AB tourette* OR AB "myocardial infarction" OR AB soil OR AB "multiple sclerosis" OR AB bipolar OR AB autism OR AB autistic OR AB ADHD	222,687
S22	TI schizophreni* OR TI "mild cognitive impairment" OR TI "brain injury"	93,703
S23	AB schizophreni* OR AB "mild cognitive impairment" OR AB "brain injury"	159,706
S24	S11 OR S12 OR S13 OR S14 OR S15 OR S16 OR S17 OR S18 OR S19 OR S20 OR S21 OR S22 OR S23	785,070
S25	S10 NOT S24	3,059
