# Supplementary figures and images for: The Impact of Free and Added Sugars on Cognitive Function: A Systematic Review and Meta-Analysis
Source: Nutrients. 2023 Dec 25;16(1):75. doi: 10.3390/nu16010075 (PMC10780393; doi:10.3390/nu16010075)

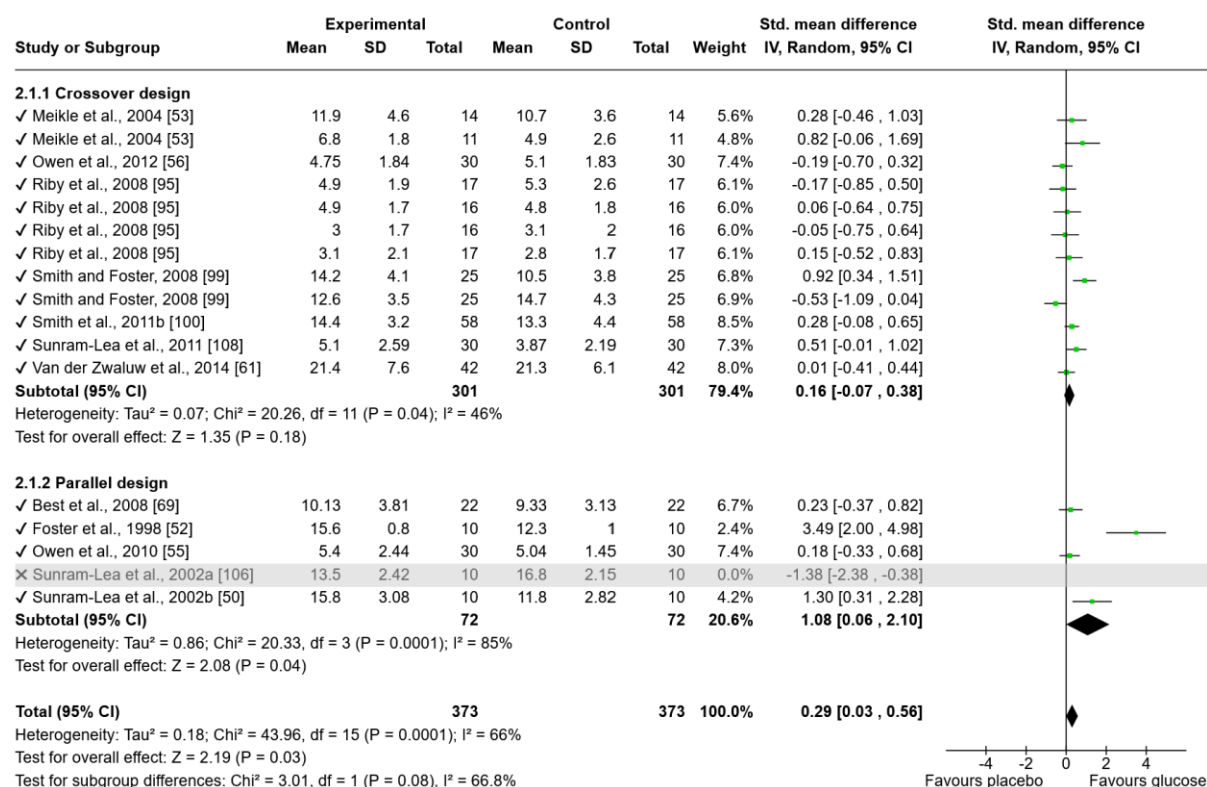

**Supplementary Figure S3: Influence analysis of sugar consumption and delayed free recall.**

Supplement: Supplementary file 1 [file nutrients-16-00075-s001.zip › Free_Sugars_Supplementary File S3_v2.pdf]
